# Machine Learning for Drug-Target Interaction Prediction

**DOI:** 10.3390/molecules23092208

**Published:** 2018-08-31

**Authors:** Ruolan Chen, Xiangrong Liu, Shuting Jin, Jiawei Lin, Juan Liu

**Affiliations:** 1 Department of Computer Science, School of Information Science and Technology, Xiamen University, Xiamen 361005, China; chenruolan@stu.xmu.edu.cn (R.C.); xrliu@xmu.edu.cn (X.L.); stjin.xmu@gmail.com (S.J.); 23020161153321@stu.xmu.edu.cn (J.L.); 2 Department of Instrumental and Electrical Engineering, School of Aerospace Engineering, Xiamen University, Xiamen 361005, China

**Keywords:** drug-target interaction prediction, machine learning, drug discovery

## Abstract

Identifying drug-target interactions will greatly narrow down the scope of search of candidate medications, and thus can serve as the vital first step in drug discovery. Considering that in vitro experiments are extremely costly and time-consuming, high efficiency computational prediction methods could serve as promising strategies for drug-target interaction (DTI) prediction. In this review, our goal is to focus on machine learning approaches and provide a comprehensive overview. First, we summarize a brief list of databases frequently used in drug discovery. Next, we adopt a hierarchical classification scheme and introduce several representative methods of each category, especially the recent state-of-the-art methods. In addition, we compare the advantages and limitations of methods in each category. Lastly, we discuss the remaining challenges and future outlook of machine learning in DTI prediction. This article may provide a reference and tutorial insights on machine learning-based DTI prediction for future researchers.

## 1. Introduction

Most drugs demonstrate efficacy via the in-vivo interactions with their target molecules such as enzymes, ion channels, nuclear receptors and G protein-coupled receptors (GPCRs). Therefore, identifying drug-target interactions (DTIs) has become a vital precondition in cognate areas including poly-pharmacology, drug repositioning, drug discovery, side-effect prediction and drug resistance [1]. The experimentation and confirmation of drug-target pairs have been great hindrances to many drug researches. On top of that biochemical experiments for undiscovered drug-target interactions involve significantly costly, time-consuming and challenging work. For instance, it takes around 1.8 billion dollars for each new molecular entity (NME) [2] as well as an average time span of 9 to 12 years for the approval of a new drug application (NDA) [3].

Besides the known interactions already stored in various databases, there exist countless unpaired small molecule compounds that could potentially be discovered and developed into new medications. Only a small number of drug-target pairs have been experimentally validated in the current data set. In fact, although there are more than 90 million compounds described in the PubChem database, a large proportion of interactions still remain to be discovered [4]. Furthermore, the number of truly innovative drugs approved by regulatory agencies has decreased in recent years, despite the progress in biotechnology. For instance, it is reported that US Food and Drug Administration (FDA) only approves approximately 20 novel drugs every year with high investment costs [5]. These large time, money and resource costs, both human and material, have motivated researchers to constantly develop innovative technology for the exploitation of new drugs. Interaction prediction helps to screen new drugs candidates effectively and efficiently.

Identifying new targets for existing or abandoned drugs, namely drug repositioning, is another important part in drug discovery. The “multi-target, multi-drug” in place of “one target, one drug” model has been widely accepted as our understanding of pharmacology deepens [1]. The important fact is that drugs typically target multiple proteins rather than only one. The anticancer drugs sunitinib (Sutent) and imatinib (Gleevec) are both concrete evidence. What’s more, drugs may interact with other proteins in addition to the primary therapeutic targets, namely off-target effects. Off-target effects are typically considered harmful side effects. However, in some cases, they may be beneficial since they could lead to unexpected therapeutic effects and provide a new perspective on the molecular mechanisms of drug side effects. The purpose of drug repositioning is the detection for new clinical uses for existing drugs. An obvious benefit of drug repositioning is that existing drugs have already been strictly verified for their safety and bioavailability. Omitting some previously completed steps can greatly speed up the drug development process. Governments, academic institutions and non-trading organizations around the world have made more effort into drug repositioning recently which will effectively facilitate the repositioning research [6].

For all the reasons mentioned above, detecting drug-target interactions is fundamental to both new drug discovery and old drug repositioning. The known drug-target interactions based on wet-lab experiments are limited to a very small number. The huge gap between known and unknown drug-target pairs has prompted interest in DTI prediction. Traditional prediction strategies in vitro have faced the limitations of time and monetary costs, while recently developed computational or in silico methods can more efficiently predict potential interaction candidates. Computational methods have achieved favorable performance in many related bioinformatics fields, such as disease-related miRNA prediction [7,8,9], disease genes prediction [10], protein-protein interaction prediction [11] and protein subcellular location prediction [12]. They greatly narrow the broad scope of research of experimental DTI validation. Therefore, there is a continuous and urgent demand for the development of computational techniques on DTI predictions.

Currently, the ligand-based, docking simulation, and chemogenomic approaches are the three main classes of computational methods for predicting DTIs. Ligand-based methods [13] like Quantitative Structure Activity Relationship (QSAR) utilize the idea that similar molecules usually bind to similar proteins. Specifically, these methods predict interactions by comparing a new ligand to known proteins ligands. However, ligand-based methods perform poorly when the number of known ligands is insufficient.

As for docking simulation methods [14], the three-dimensional (3D) structures of proteins are required for simulation hence becoming inapplicable when there are numerous proteins with unavailable 3D structures. Moreover they cannot be applied to membrane proteins like ion channel and G-Protein Coupled Receptors (GPCRs) whose structures are too complex to obtain. Docking simulations usually take significant time and thus it can be especially inefficient.

**Figure 1:**
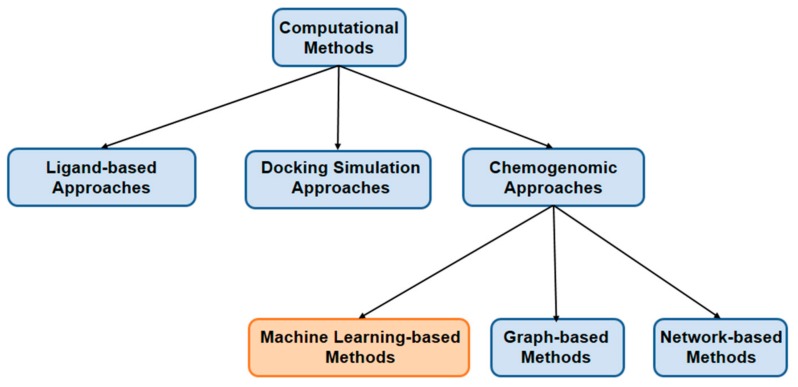
Branch diagram of recent computational methods for DTI prediction.

To address the difficulties of traditional methods, chemogenomic approaches [15] have recently been performed successfully in drug discovery and repositioning on a large scale. There are four main types of target frequently involved in DTI prediction, namely protein, disease, gene and side effect. For the purpose of drug-target pair prediction, these methods integrate both the chemical space of compounds and the genomic space of target proteins into a unified space: pharmacological space. Hence, chemogenomic approaches can make full use of abundant biological data that is favorable for prediction. In such a DTI prediction problem, the major challenge is the scarcity of known drug-protein interactions and unverified negative drug-target interaction samples. These chemogenomic approaches can be classified into different categories, such as machine learning-based methods, graph-based methods and network-based methods [16]. Among all the chemogenomic approaches, machine learning-based methods have gained the most attention for their reliable prediction results. Most of these methods generally utilize the chemical and biological features of drugs and targets, and adopt various machine learning techniques to predict interactions between drugs and targets. Figure 1 is a branch diagram of recent computational methods for DTI prediction.

In this review, we focus on machine learning methods applied to DTI prediction. To be specific, we aim to provide a comprehensive overview on a subclass of chemogenomic approaches exploiting machine learning frameworks. Compared with those ligand-based methods that also apply machine learning strategies, the methods discussed in this review can be applicable to target proteins with insufficient known ligands. Firstly, we summarize a brief list of databases frequently used in drug discovery. Next, we adopt a hierarchical classification scheme. In particular, we classify the machine learning methods into two major categories i.e., supervised and semi-supervised methods, and provide more subclasses. We attempt to introduce several representative methods of each category, respectively. Furthermore, we present the advantages and disadvantages for methods of each category. Finally, we will discuss the challenges and further outlook for current machine learning methods in DTI prediction domain from our point of view.

1. Supervised Learning Methods
  Both positive labels and negative labels are required in the training set. Then these labeled samples are used to train the learning models for subsequent DTI prediction.
  - Similarity-based methods
    The similarities among drugs or among targets are calculated via various similarity measurement strategies. Similarity matrices can be utilized in various types of kernel functions:
    (i) The nearest neighbor methods: The nearest neighbor methods make predictions based on the information of the nearest neighbors.
    (ii) Bipartite local models: Two local models are firstly trained for drugs and targets respectively. The final prediction result for each drug-target pair is computed based on the operation of the two independent prediction scores.
    (iii) Matrix factorization methods: Drug-target interaction matrix is factorized into two latent feature matrices that when multiplied together can approximate the original matrix.
  - Feature vector-based methods
    The training data is represented as feature vectors. Then some machine learning models, like Random Forest, can be utilized for prediction based on these vectors.
2. Semi-Supervised Learning Methods
  Semi-supervised learning methods make predictions only based on a small amount of labeled data and a large amount of unlabeled data.
  To our best knowledge, there are already some excellent reviews on chemogenomic approaches for DTI prediction [6,15,16,17,18,19]. Compared to previous works, we focus on the special topic of machine learning methods used in DTI prediction. Besides, we utilize a hierarchical classification scheme and summarize several latest prediction methods such as [20,21,22,23] which are hardly mentioned in any previous review. In particular, review [17] is written only from a narrow viewpoint, namely similarity-based approaches, which are a subclass of machine learning methods. Surveys [6,15,18,19] all provide a more general and comprehensive overview of chemogenomic approaches rather than emphasizing machine learning. In recent years, machine learning has made breakthroughs and attracted a lot of public attention. Discussing state-of-the-art DTI prediction strategies from this special perspective can demonstrate more methodology details. Although review [16] also focuses on learning-based methods, its emphasis is only on supervised learning. In comparison, we provide more detailed sub-classes and introduce newly developed methods after review [16] was published.
  The rest of this article is organized as follows: The “Databases” section describes current available data sources for DTI prediction research. The “Methods” section briefly introduces several representative machine learning methods via a hierarchical classification scheme. Then we discuss advantages and limitations of methods in each category as well as remaining challenges. Finally, the “Conclusions and Outlook” section makes a future perspective for machine leaning in DTI prediction.

## 2. Databases

Data mining and utilization based on the existing bioinformatics databases is a significant methodology for drug discovery. With the development of molecular biology, abundant information about drugs and targets has accumulated. Thus, it is necessary to establish databases for managing and maintaining the data. There exist a number of different professional databases involving potential cellular targets for various families of chemical compounds up to now. A large portion of them are publicly available. Moreover, the data size is increasing owing to the contributions of researchers from around the world. As more information about drugs and targets is collected, there are more opportunities for drug discovery research. To a certain degree, these databases have promoted the development of latest methodologies for drug discovery. In Table 1, we list frequently used databases, their web servers and brief descriptions. Table 2 shows the statistics of the number of compounds, targets and compound-target interactions in these databases. Note that not all databases provide complete information in their databases and published papers.

Some of these databases are being updated frequently, such as DrugBank, KEGG, and STITCH and so on, while the data in other databases has remained almost the same for several years, such as SuperPred which was last updated in April 2014. It is, however, encouraging that more new databases and easy-to-use web servers have been recently established. On one hand, the existing databases provide plentiful data sources of drug space and target space. It is time for the researchers to make efforts to integrate more different types of heterogeneous data. On the other hand, current databases do not involve any non-interaction information. This common drawback has limited the prediction result of supervised learning methods. Thus it would be meaningful to make public both interactions and non-interactions between drugs and targets in the future.

## 3. Methods

In the era of big data, machine learning methods are designed to generate predictive models based on some underlying algorithm and a given big data set. For biological and biomedical research, machine learning plays a pivotal role in filtering large amounts of data into patterns [24,25,26,27]. The general machine learning workflow in DTI prediction can be divided into three steps. First, preprocessing the input data of the drug and the target; second, training the underlying model based on a set of learning rules; third, utilizing the predictive model to make predictions for a test data set.

From our research, study [28] is the first work that applies machine learning to protein-chemical interaction prediction. This work establishes a SVM analysis framework of amino acid sequence data, chemical structure data and mass spectrometry data. This pioneering study has inspired subsequent studies. Machine learning for drug discovery has become a field of long-standing and growing interest since then.

For simplicity, we classify machine learning methods for drug-target interaction prediction into two major categories, i.e., supervised learning and semi-supervised methods. Specifically, the supervised learning methods can be further classified into two sub-classes including similarity-based methods and feature-based methods. 

### 3.1. Supervised Learning Methods

Supervised learning methods are applied to train the learning model and identify patterns when labels are available. For the DIT prediction problem, known drug-target interactions are labeled as positive samples and the rest are labeled as negative ones. Next, these labels are used to train the model for subsequent interaction predicting. In fact, those drug-target pairs without explicit interaction information may correspond to unknown or missing interactions rather than non-interactions. In general results of non-interactions between drugs and targets are not published. Methods of this category regard all the unknown drug-target interactions as non-interaction despite inaccuracy. In the section, we will review the supervised methods proposed so far in two categories, i.e., similarity-based methods and feature-based methods.

#### 3.1.1. Similarity-Based Methods

A key underlying assumption of similarity-based machine learning methods is the “guilt-by-association” assumption, that is, similar drugs tend to share similar targets and vice versa. In this kind of approach, the similarity among drugs or among targets is computed by various similarity measures. The constructed similarity matrices define several types of kernel functions.

• The Nearest Neighbor Methods

The nearest neighbor methods generally adopt relatively simple similarity functions. Researchers often integrate these methods with some other approaches to help predict new drugs or targets, such as models in paper [46,47]. In the early stage, study [48] proposed two exploratory approaches, namely the nearest profile method (NN) and the weighted profile method. The nearest profile method follows the key concept that similar drugs or targets tend to be close in the network. This method was used in [49] as the baseline. In contrast, the weighted profile method utilizes the similarities of all the other drugs and targets and then adopts a weighted average. However, these methods show poor performance in the case when targets bound to similar drug share low sequence similarity or vice versa.

In the studies [23,50] by Zhang et al., methods that make drug-drug pair predictions based on neighbors were developed. These studies further extended the classic neighbor recommender method to the integrated neighborhood-based method (INBM). In simple terms, neighbor recommender method generally uses the weighted average information of neighbors for prediction. INBM is an ensemble model that integrates several neighborhood-based models for a robust prediction. For each drug-drug pair, three commonly used formulas, namely Jaccard similarity, Cosine similarity and Pearson correlation similarity, are used to calculate similarity score.

Another novel methodology in this category is Similarity-Rank-based predictor (SRP) [51]. Two indices, i.e., tendency index and inverse tendency index, are computed to construct a SRP. To be specific, the former represents the likelihood that each drug–target pair tends to interact, while the latter measures the tendency that each drug–target pair does not interact. The calculation formulas involve both similarity and similarity rank. Then an interaction likelihood score is computed as the likelihood ratio of the two indices. This method can generate two interaction likelihood scores, one from the drug side and the other from the target side. The final prediction score is the average of the two scores. The clear advantage of SRP is that it is a lazy and non-parametric model without the requirements of an optimization solver, prior statistical knowledge as well as tunable parameters.

In recent years, other new similarity-based methods have been proposed one after another, such as rule-based inference. Due to the limitation of the previous topology-based methods, a similarity-based deep learning method [52] merges the similarity measure with two rule-based inference methods. In other words, drug-based similarity inference (DBSI) and target-based similarity inference (TBSI) [48,53] are adopted to discover the drug-target interactions with the similarities. Though it is flexible to assemble any kernel functions, the method cannot predict new drugs or targets.

Note that most of similarity measures only utilize some important drug-related or disease-related properties to perform drug-disease prediction and ignore the known drug-disease interaction information [54]. Some researchers have proposed new similarity measures. Luo et al. [54] have designed a comprehensive similarity measure. In order to improve traditional similarity measures for drug-disease prediction, the comprehensive similarity measure has integrated drug or disease feature information with known drug–disease interactions. The similarity measure can be broken down into three steps. In the first step, drug similarity and disease similarity are calculated based on drug-related properties or disease-related properties respectively. In the second step, these similarity values are adjusted by a logistic function based on the analysis and evaluation results. In the last step, a weighted drug network can be established for the drug similarity. The edge weight represents the number of common diseases between corresponding drugs. Then a cluster method, ClusterONE, is applied to identify potential drug clusters. Similarity between drugs belonging to the same cluster is enhanced and thus comprehensive drug similarity is obtained. Disease similarity can be improved in the same way as for drugs.

• Bipartite Local Models

Bipartite local models (BLMs) firstly generate two independent prediction for drugs and targets respectively. The final prediction result is then obtained by aggregating the two prediction scores.

The concept of BLM was first introduced in the pioneering work by Bleakley and Yamanishi [49]. This method can transform the drug-target interaction prediction problem into a binary classification problem. More specifically, a local model is trained for drugs based on chemical similarity. Another one is trained for proteins based on sequence structure. Therefore, two SVM classifiers can generate two independent prediction results from the drug or target side respectively. Final prediction result for each drug-target pair is computed based on the average of these two independent prediction scores.

Analogously, another method [55] developed a regularized least square classifier introducing two algorithms, called RLS-avg and RLS-kron. In particular, Regularized Least Squares (RLS-avg) utilizes kernel ridge regression to perform prediction. While in RLS-kron, all pairs of drugs and targets are combined into one to make Kronecker product, bringing the runtime down greatly.

Considering the limitation of the BLM-based methods above of predicting new drug or target without any known interactions available, Mei et al. [46] extended existing BLM by adding a preprocessing to infer training data from neighbors’ interaction profiles. The method is called Bipartite Local Models with Neighbor-based Interaction Profile Inferring (BLM-NII). BLM-NII involves RLS-avg algorithm and is proven to be effective in new candidate problem.

• Matrix Factorization Methods

Matrix factorization methods are typically used in recommendation systems to find potential user-item interactions. The DTI prediction can be regarded as a matrix completion problem that aims to look for missing interactions. Therefore, drug-target interaction matrix can be factorized into two other matrices that when multiplied together can approximate the original matrix.

Kernelized Bayesian Matrix Factorization with Twin Kernels (KBMF2K) [56] is the original method that introduced matrix factorization to DTI prediction. Following some previous approaches, KBMF2K defines two kernel matrices only based on chemical similarity between drug compounds and genomic similarity between target proteins. It combines Bayesian probabilistic formulation, matrix factorization and binary classification for prediction problem.

Another study adopting probabilistic formulations is Probabilistic Matrix Factorization (PMF) [57]. PMF is distinguished greatly from KBMF2K by its independence of drug or target similarity matrices. Furthermore, the study presented the active learning (AL) strategy along with probabilistic matrix factorization.

Zheng et al. [58] proposed an extension of weighted low-rank approximation from one-class collaborative filtering (CMF), namely Multiple Similarities Collaborative Matrix Factorization (MSCMF). MSCMF integrates multiple similarity matrices, including chemical structure similarity, genomic sequence similarity, ATC similarity, GO similarity and PPI network similarity. Weights over the matrices are estimated to select similarities automatically. This strategy improves predictive performance in the experiment. Drugs and targets are projected into low-rank matrices. Then weights over similarity matrices are estimated using an alternating least squares algorithm. However, regardless of its performance, under this data integration strategy, a large amount of information may be lost, thus leading to sub-optimal solution.

The method developed by Ezzat et al. [59], employed two matrix factorization methods (i.e., GRMF and WGRMF). It was revealed in previous work [60] that data usually lies on or nears to the low-dimensional and non-linear manifold. Therefore, GRMF and WGRMF perform manifold learning implicitly by means of graph regularization. In addition, a preprocessing step (WKNKN) was applied to new drug or target prediction by transforming all the 0’s in the original drug-target matrix into interaction likelihood values. This important step distinguishes this method from other work that regards all the 0’s of given drug-target matrix as non-interaction roughly, and thus enhances the prediction results.

#### 3.1.2. Feature Vector-Based Methods

Generally, similarity-based prediction algorithms do not take heterogeneous types and interactions defined in semantic networks into consideration. In addition, it may be difficult to add the long indirect connections between two nodes. Therefore, feature vector-based methods have been utilized for DTI prediction. The input of feature vector-based methods is drug-target pairs represented by fixed-length feature vectors. The feature vectors are encoded by various properties of drugs and targets.

In the systematic approach [61], chemical descriptors are calculated using DRAGON program (http://www.talete.mi.it/index.htm). Finally, each drug is represented as a set of 1080 descriptors, including constitutional descriptors, topological descriptors, 2D autocorrelations, eigenvalue-based indices and so on. Likewise, each protein is represented by a set of structural and physicochemical descriptors via PROFEAT WEBSEVER (http://jing.cz3.nus.edu.sg/cgi-bin/prof/prof.cgi). The descriptors involve Amino acid composition descriptors, Dipeptide composition descriptors, and Autocorrelation descriptors and so on. Then each protein sequence with changeable length can be transformed into a standard feature vector of 1080 dimensions. Hence, a set of 2160-dimensional feature vectors for each drug-target pair can be constructed. Subsequent prediction step performs Random Forest (RF) algorithm which introduces random training set (bootstrap) and random input vectors into the trees. The comprehensive framework shows its robustness against the over fitting problem and performs more efficiently for a large-scale data set in experiments.

In order to integrate diverse information from heterogeneous data sources, a method named DTINet was proposed by Luo et al. [20]. Through DTINet, a low dimensional feature vector that accurately explains the topological properties of each node in the heterogeneous network is first learned. In the further step, DTINet applies inductive matrix completion to best project drug space onto protein space.

Due to the fact that DTINet separates features and may result in loss of the optimal solution, Wan et al. [21] created a new framework called neural integration of neighbor information for DTI prediction (NeoDTI). The inspiration of NeoDTI came from convolution neural networks (CNNs). It integrates the neighbor information in heterogeneous network. After extracting the complex hidden features vectors of drugs and targets, NeoDTI automatically learns topology-preserving representations to achieve superior prediction performance.

The pioneering effort in [62] introduced a two-layer undirected graphical model, namely restricted Boltzmann machine (RBM), into a large-scale drug-target interaction prediction. There are no intra-layer connections in these layers. What’s more, RBM model is trained via a practical learning algorithm, i.e., Contrastive Divergence (CD). Where the method significantly outperforms other existing approaches is in that it can predict different types of DTIs on a multidimensional network. In other words, the method can identify binary DTIs as well as their corresponding types of interactions, including relationships and drug modes of action.

In the paper published by Fu and cooperators [63], a state-of-the-art machine learning model was constructed based on meta-path-based topological features. Two measures of topological features are calculated, including the number of path instances between nodes and a normalization process to it. Given features, a Random Forest algorithm is used as supervised classification. Furthermore, intrinsic feature ranking algorithm embedded in Random Forest selects the important topological features for better prediction. This framework has shown precise predictability.

### 3.2. Semi-Supervised Learning Methods

Considering the negative sample selection has a great influence on the accuracy of DTI prediction results, some researchers have proposed semi-supervised methods to address the problem. These methods use only a small amount of labeled data and a large amount of unlabeled data. Semi-supervised methods typically use the labeled data to infer labels for unlabeled data. On the other hand, the unlabeled data can also help provide insights into the structure of training set.

Having no use of negative samples, study [64] first employed a manifold Laplacian regularized least square (LapRLS) based on the BLM concept. Furthermore, an extension of the standard LapRLS, namely NetLapRLS, was proposed. NetLapRLS integrates information from chemical space, genomic space and drug-protein interaction for a new kernel. These semi-supervised methods have achieved encouraging results than using the labeled data alone. However, it is time-consuming when implementing them on a large scale.

Another method is designed for both semi-supervised and unsupervised settings. Ma et al. [22] presented a new framework to learn accurate and interpretable similarity measures when labels are scarce. This framework constructs a set of Graph Auto-Encoder (GAE)-based models and integrates multi-view drug similarities. Besides, an attentive mechanism is used for view selection and better interpretability.

### 3.3. Discussion

Each machine learning model possesses its unique advantages as well as disadvantages. Note that just as the popular concept in computer science, namely “no free lunch theorem” [65], machine learning methods are context-specific. Therefore, in this review we can only evaluate the advantages and disadvantages of each method category based on DTI prediction context.

A number of supervised models have been already proven feasible for DTI prediction. However, most supervised methods simply regard all the unlabeled drug-target pairs as negative samples and thus generate inaccurate predictive results. What’s more, each similarity-based method has its limitation when extending to large a data set because of high complexity of similarity matrices computation.

Consider the three sub-classes of similarity-based methods respectively. Although the nearest neighbor methods generally apply relatively simple similarity functions, most of them construct neighborhoods only based on first-order similarity and do not involve the transitivity of similarity [66]. A key advantage of bipartite local models is that they process much fewer drug-target pairs, and thus they have much lower complexity than global models. Nevertheless, bipartite local models cannot handle the scenario that both drugs and targets are not involved in the training set unless combined with other methods. According to the experiment result in [19], matrix factorization methods generally have more superior performance than other methods including the nearest neighbor models and bipartite local models.

A small number of known drug-target interactions results in an imbalanced dataset. As an effective solution for imbalanced datasets, semi-supervised learning uses only a small amount of labeled data with a large amount of unlabeled data and generates more reliable prediction than supervised one.

In addition to the aforementioned single machine learning methods, we also have introduced several ensemble methods [61,63]. A better and robust prediction generally results from the biases trade-off of each single method. Generally, ensemble methods can combine different learning models. For more ensemble methods applied to drug-target interaction prediction task, please refer to [67,68,69].

Generally, machine learning has achieved favorable performance in DTI prediction. Nonetheless, a number of challenges still remain. Above all, recently, some researchers have emphasized that predictive models based on machine learning are usually established and evaluated with overly simplified settings. Prediction results under such experiment settings may be over optimistic and deviate from the real case. Particularly, most of machine learning methods simply regard drug-target interaction as an on-off relationship and ignore other vital factors like molecule concentrations and quantitative affinities. Pahikkala et al. [24] have pointed out four factors having significant impact on prediction results, including problem formulation, evaluation data set, evaluation procedure and experimental setting. Considering the binding affinities and dose-dependence of drug-target pairs, the DTI prediction problem should be formulated as a regression or rank prediction problem rather than a standard binary classification problem. The second challenge is the imbalanced dataset problem. Due to the small number of known drug-target pairs, the current dataset is imbalanced. Some models like decision trees and SVMs, have a great bias for recognizing the majority class and thus result in poor performance [16]. Thirdly, most machine learning models possess “poor interpretability” properties. In other words, it is difficult to understand the underlying drug mechanism of action from a biological perspective. Note that in most case, it is easier to explain relatively simple models. This case is consistent with one of the “rules of thumb” [70], that is “simple is often better”. Nonetheless, for most current state-of-the-art approaches achieving high DTI prediction accuracy, such as deep learning methods, it is difficult to interpret them from a pharmacology perspective. Last but not least, there are still no uniform evaluation metrics special for DTI prediction. Previous studies have adopted some common evaluation metrics in bioinformatics [71], such as sensitivity, specificity, Area Under the Precision-Recall (AUPR) curve and Area under the ROC curve (AUC). The fact is that if the sensitivity increases, the specificity decreases. Considering the limitation of using sensitivity or specificity alone, AUPR and AUC may be better choices in evaluation tasks. In the currently accessible datasets, the number of unknown samples is much more than the known ones, and thus false positives should be weighed more. AUPR can reduce the impact of false positive data on evaluation results as possible [72], and AUC is insensitive to imbalance dataset [73]. Thus both AUPR and AUC are generally adequate metrics for evaluating the performance of machine learning-based methods.

## 4. Conclusions and Outlook

DTIs contribute to the selection of potential drugs and thus effectively reduce the scope of research for biochemical experiments. Besides, they can provide deep insights into the side effects and the mechanism(s) of action of drugs. Hence, DTI prediction is a vital prerequisite for drug discovery. In fact, a number of public available databases have been established and promoted the development of innovatory DTI prediction strategies.

In this review, we focus on machine learning-based methods integrating chemical space and genomic space. We summarize the databases and machine learning methods frequently used in DTI prediction. In particular, we focus on several state-of-the-art predictive models appearing in recent years. We adopt a hierarchical classification scheme. We classify machine learning methods into two major categories: supervised and semi-supervised methods, and provide more subclasses.

Machine learning will be promising in DTI prediction for the next several years. However, there is still much room for improvement. Hence, we conclude with some advice as a reference for the future researchers.

Firstly, ensemble approaches combine multiple independent classifiers into one model and typically achieve a better prediction results. Next, semi-supervised learning is a powerful tool for addressing the imbalanced dataset problem. However, only a small number of semi-supervised learning methods have been proposed recently. Hence, the research on semi-supervised learning methods needs more attention. Furthermore, note the fact that drug-target pairs involve binding affinities and dose-dependence. It is more practical and meaningful to study new regression methods for DTI prediction problem. The using of quantitative bioactivity data will lead to a more accurate and reliable predictive result. Finally, with the development of high throughput biotechnology, the available data has been growing quickly recently. It is time for further machine learning technology to take full advantage of more different types of heterogeneous data.

## 5. Key Points

1. Identifying drug-target interactions is the vital first step in drug discovery research.
2. A number of existing professional databases serve known data resources for DTI prediction and thus promote the drug discovery.
3. Machine learning-base methods are generally effective and reliable for DTI prediction.
4. Different machine learning methods have their merits and demerits. Hence, it is essential to choose appropriate methods or assemble models for special prediction tasks.
5. A more effective prediction model can be established by integrating more heterogeneous data sources of drugs and targets.
6. In reality, DTI prediction is a regression problem with quantitative bioactivity data.

## Figures and Tables

**Table 1:** Databases supporting drug discovery methods.

Database and URL	Brief Descriptions
KEGG [29] http://www.genome.jp/kegg	An encyclopedia of genes and genomes for both functional interpretation and practical application of genomic information.
BRENDA [30] http://www.brenda-enzymes.org/	The main enzyme and enzyme-ligand information system.
PubChem [31] https://pubchem.ncbi.nlm.nih.gov/	A database for information on chemical substances and their biological activities involving three inter-linked databases, i.e., Substance, Compound and BioAssay.
TTD [32] http://bidd.nus.edu.sg/group/ttd/ttd.asp	Therapeutic Target Database providing comprehensive information about the drug resistance mutations, gene expressions and target combinations data.
DrugBank [33] http://www.drugbank.ca	Consisting of two parts information involving detailed drug data (i.e., chemical, pharmacological and pharmaceutical) and drug target information (i.e., sequence, structure, and pathway) respectively.
SuperTarget [34] http://bioinf-apache.charite.de/supertarget	A database integrating drug-related information with more than 330,000 compound-target protein relations.
ChEMBL [35] https://www.ebi.ac.uk/chembldb	Data resource for molecule structures and molecule-protein interactions collected from the primary published literature on a regular basis.
STITCH [36] http://stitch.embl.de/	Repository of known and predicted chemical-protein interactions.
MATADOR [37] http://matador.embl.de/	A database of protein-chemical interactions including as many direct and indirect interactions as possible.
BindingDB [38] http://www.bindingdb.org/bind	A public database of protein-ligand binding affinities.
TDR targets [39] http://tdrtargets.org/	A chemogenomics resource for neglected tropical diseases.
SIDER [40] http://sideeffects.embl.de/	Serving information on marketed medicines and their recorded adverse drug reactions.
ChemBank [41] http://chembank.broad.harvard.edu/	Collections of available data derived from small molecules and small-molecule screens and resources for studying their properties.
DCDB [42] http://www.cls.zju.edu.cn/dcdb/	The Drug Combination Database for collecting and organizing known examples of drug combinations.
CancerDR [43] http://crdd.osdd.net/raghava/cancerdr/	Cancer Drug Resistance Database of 148 anticancer drugs and their effectiveness against around 1000 cancer cell lines.
ASDCD [44] http://asdcd.amss.ac.cn/	The first Antifungal Synergistic Drug Combination Database including published synergistic antifungal drug combinations, targets, indications, and other pertinent data.
SuperPred [45] http://prediction.charite.de/	Resource of compound-target interactions.

**Table 2:** The statistics of the number of compounds, targets and compound-target interactions in the databases covered in the review.

Databases	The Number of Compounds	The Number of Targets	The Number of Compound-Target Interactions
KEGG	18,380	26,885,475	
BRENDA		7341	
PubChem	96,479,316	68,868	
TTD	34,019	3101	
DrugBank	11,682	26,889	131,724
SuperTarget	195,770	6219	332,828
ChEMBL	2,275,906	12,091	
STITCH	500,000	9,600,000	1,600,000,000
MATADOR	775		
BindingDB	652,068	7082	1,454,892
TDR targets	2,000,000	5300	
SIDER	5868	1430	139,756
ChemBank	1,700,000		
DCDB	904	805	
CancerDR	148	116	
ASDCD	105	1225	210
SuperPred	341,000	1800	665,000
